# Effect of Metformin on Vitamin B12 Deficiency in Patients With Type 2 Diabetes Mellitus and Factors Associated With It: A Meta-Analysis

**DOI:** 10.7759/cureus.32277

**Published:** 2022-12-07

**Authors:** Yasitha Kakarlapudi, Sai Kiran Kondabolu, Zuha Tehseen, Vimal Khemani, Srilakshmi K J, Maira D Nousherwani, Faraz Saleem, Ahmed N Abdelhameed

**Affiliations:** 1 Internal Medicine, Andhra Medical College, Visakhapatnam, IND; 2 Internal Medicine, Allama Iqbal Medical College, Lahore, PAK; 3 Internal Medicine, Jinnah Sindh Medical University, Karachi, PAK; 4 Paediatrics, Dr B R Ambedkar Medical College and Hospital, Kochi, IND; 5 Medicine, Shalamar Institute of Health Sciences, Lahore, PAK; 6 Internal Medicine, Akhtar Saeed Medical and Dental College, Lahore, PAK; 7 Faculty of Medicine, Al-Azhar University, Asyut, EGY

**Keywords:** meta-analysis, type 2 diabetes, factors, vitamin b12, metformin

## Abstract

The current meta-analysis aims to explore the effect of metformin use on vitamin B12 deficiency in patients with type 2 diabetes mellitus (T2DM) and the factors associated with it. This meta-analysis followed the Meta-analysis Of Observational Studies in Epidemiology (MOOSE) guidelines and the Preferred Reporting Items for Systematic reviews and Meta-Analysis (PRISMA) guidelines. We searched PubMed and EMBASE from January 1, 2010, to October 31, 2022, to collect the studies that reported the effect of metformin on the deficiency of vitamin B12 in patients with T2DM and the factors associated with it. A total of 17 studies were included in the current meta-analysis. Among all the included studies, 13 were cross-sectional studies, 3 were retrospective cohorts, and one was a case-control study. The pooled rate of deficiency of vitamin B12 in patients receiving metformin (23.16%) was significantly higher compared to patients who were not on metformin (17.4%) (OR: 2.95, 95% CI: 2.18-4.00, p-value: 0.001). Factors significantly associated with vitamin B12 deficiency in patients with T2DM and receiving metformin include the duration of metformin use and a greater dose of metformin. In conclusion, our meta-analysis found that the prevalence of vitamin B12 deficiency is greater in patients receiving metformin compared to patients who did not receive metformin. Given the importance of vitamin B12 in nutrition, metformin-induced B12 decrease may be harmful to patients with T2DM. Supplemental vitamin B12 may be advantageous for those on metformin.

## Introduction and background

Type 2 diabetes mellitus (T2DM) is associated with significant mortality and morbidity, with the potential to adversely affect the neurologic, renal, and cardiovascular systems of individuals with the disease [1]. Treatment of T2DM includes certain options like medications and lifestyle changes. The biguanide medicine metformin is a drug that has shown promise in the treatment of type 2 diabetes and is advised as the first choice for oral management by the American Diabetes Association [2]. Metformin has positive impacts on vascular protection, weight loss, and carbohydrate metabolism [3] but long-term use of metformin was found to be associated with the risk of anemia [4]. It is reported that 30% of patients receiving long-term metformin experience vitamin B12 malabsorption, with a reduction in the concentration of serum vitamin B12 of 14% to 30% [5].

Vitamin B12 is an important nutrient for health. It can play a significant role in the formation of red blood cells and the functioning of the nervous system and brain. A deficiency of vitamin B12 may increase the severity of diabetic neuropathy [6]. Deficiency of vitamin B12 results in methylmalonyl-CoA accumulation that is subsequently converted to methylmalonic acid (MMA), whose levels in plasma are often high in individuals with a deficiency of vitamin B12. In individuals with a deficiency of vitamin B12, enhanced levels of homocysteine and MMA have been suggested to myelopathy leading to autonomic and peripheral neuropathy [7].

About 50% of individuals with T2DM will develop a certain degree of diabetic neuropathy throughout their life [8]. This group of patients has impaired clinical suspicion of vitamin B12 deficiency based on neurological symptoms, which may result in an underdiagnosis of this serious vitamin deficit. If this condition is not properly diagnosed and treated, this can result in long-term neurological impairment [9]. Because large numbers of individuals with T2DM use metformin, the execution of a universal screening routine can enhance costs for the healthcare system [10]. Thus, identification of the risk factors associated with the development of deficiency of vitamin B12 is a way to facilitate the screening of high-risk patients, leading to the development of a more economical screening system.

The current meta-analysis explored the different factors associated with vitamin B12 deficiency in patients with T2DM and receiving metformin. To develop interventions that can help prevent deficiency of vitamin B12 in patients with T2DM and receiving metformin, understanding of factors affecting it is important. Our objective was to perform a meta-analysis of the published literature on the association between metformin use and vitamin B12 deficiency and the factors associated with it in patients with T2DM.

## Review

Methodology

This meta-analysis followed the Meta-analysis Of Observational Studies in Epidemiology (MOOSE) guidelines and the Preferred Reporting Items for Systematic reviews and Meta-Analysis (PRISMA) guidelines.

Search Strategy

We searched PubMed and EMBASE from January 1, 2010, to October 31, 2022, to collect the studies that reported the effect of metformin on the deficiency of vitamin B12 in patients with T2DM and factors associated with it. We combined Medical Subject Headings (MeSH) and free text words related to metformin, vitamin B12 deficiency, and type 2 diabetes mellitus to search the aforementioned electronic databases. A reference list of included articles and relevant reviews were manually searched for additional eligible articles.

Study Selection

Two reviewers independently searched for relevant articles. After filtering duplicates, the titles and abstracts of the remaining articles were reviewed. Full text of all eligible articles was obtained and assessed for eligibility criteria. The studies were included in the current meta-analysis if they fulfilled the following criteria: 1) observational studies assessing the effect of metformin on vitamin B12 deficiency in patients with T2DM; 2) investigated possible risk factors associated with vitamin B12 deficiency in patients with T2DM and receiving metformin, irrespective of sample size; 3) published in the English language; 4) published in 2010 or onwards. Case studies, qualitative studies, editorials, and conference abstracts were excluded.

Data Extraction

Two reviewers independently extracted data using a pre-designed data extraction form developed on a Microsoft Excel spreadsheet. Data extracted included the author's name, year of publication, and sample size. The extracted data were cross-checked, and a third reviewer was invited to arbitrate disagreements.

Risk of Bias Assessment

Two reviewers assessed the risk of bias of the included study independently using the Agency for Healthcare Research and Quality (AHRQ) methodology checklist. The AHRQ methodology checklist comprises 11 items. Each item is scored “1” when answered “Yes” and “0” when answered “No” or “Unclear.” Studies are rated as low, moderate, or high risk of bias when the total quality score is 0-3, 4-7, and 8-11, respectively. Any disagreement between two authors was resolved through discussion or involvement of a third author if required.

Data Analysis

Analysis was conducted using Comprehensive Meta-Analysis software (v 4.0 Biostat, Englewood, New Jersey) and Review Manager (RevMan) version 5.4.0 (The Nordic Cochrane Centre, The Cochrane Collaboration, Copenhagen, Denmark). Estimates of odds ratio (OR) and 95% confidence interval (CI) from each study were combined in separate meta-analysis models for each factor assessed in the current meta-analysis using a random or fixed effect model. A p-value of less than 0.05 was considered significant. Heterogeneity was assessed using I-square. Statistical heterogeneity was assessed with Cochran Q, which tests for between-study statistical variation, and I2. A p-value of less than 0.1 was considered significant for heterogeneity.

Results

**Figure 1:**
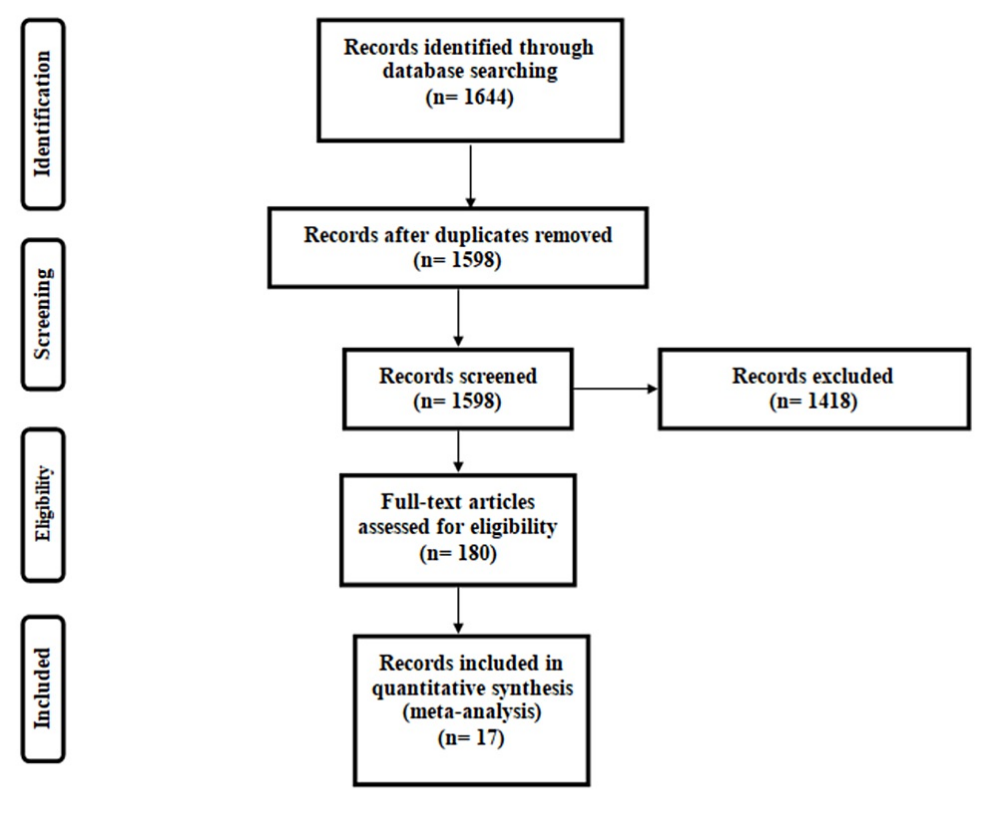
PRISMA flowchart of selection of studies PRISMA: Preferred Reporting Items for Systematic reviews and Meta-Analysis

Figure 1 summarizes the process of selection of studies. A total of 1644 articles were identified through online database searching. After removing duplicates, we reviewed the titles and abstracts of 1598 articles and identified 180 possible eligible studies for full-text screening. Among all the eligible studies, 17 were included in the current meta-analysis [11-27]. Among all the included studies, 13 were cross-sectional studies, 3 were retrospective cohorts, and 1 was a case-control study. Table 1 shows the characteristics of all included studies. The mean age of participants ranged from 49.5 years to 80.1 years. The percentage of males in each study ranged from 22.9% to 78.0%. Table 2 shows the characteristics of the included studies. Among all included studies, two were of high quality while all others were of moderate quality.

**Table 1:** Characteristics of included studies

Author Name	Year	Study Type	Sample Size	Age in Years	Males (%)
Ahmed et al [11]	2016	Cross-sectional	121	58.5	45.5
Alharbi et al [12]	2018	Retrospective cohort	412	57.2	47.6
Almatrafi et al [13]	2022	Cross-sectional	206	NR	41.3
Atabi et al [14]	2021	Cross-sectional	120	50	30.0
Damiao et al [15]	2015	Cross-sectional	231	61.7	22.9
Fakkar et al [16]	2022	Cross-sectional	100	53.3	29.0
Gundu et al [17]	2021	Cross-sectional	258	NR	46.5
Iftikhar et al [18]	2013	Case-control	219	56.5	58.4
Kim et al [19]	2019	Cross-sectional	1111	59.7	58.1
Ko et al [20]	2014	Cross-sectional	799	59	44.3
Liu et al [21]	2011	Cross-sectional	134	80.1	41.0
Martin et al [22]	2021	Retrospective cohort	13489	NR	47.6
Sato et al [23]	2013	Cross-sectional	84	62	78.6
Shahwan et al [24]	2018	Cross-sectional	200	49.5	51.0
Singh et al [25]	2013	Cross-sectional	136	51	56.6
Wai et al [26]	2018	Retrospective cohort	507	81.4	42.6
Yakubu et al [27]	2019	Cross-sectional	196	50.4	30.1

**Table 2:** Risk of quality assessment Component 1: Define source of information (survey, record, review); Component 2: List inclusion and exclusion criteria for exposed and unexposed subjects (cases and controls) or refer to previous publications; Component 03: Indicate time period used for identifying patients; Component 4: Indicate whether or not subjects were consecutive if not population-based; Component 5: Indicate if evaluators of subjective components of study were masked to other aspects of the status of the participants; Component 6: Describe any assessments undertaken for quality assurance purposes (e.g., test/retest of primary outcome measurements); Component 7: Explain any patient exclusions from analysis; Component 8: Describe how confounding was assessed and/or controlled; Component 9: If applicable, explain how missing data were handled in the analysis; Component 10: Summarize patient response rates and completeness of data collection; Component 11: Clarify what follow-up, if any, was expected and the percentage of patients for which incomplete data or follow-up was obtained.

Author Name	Component 1	Component 2	Component 3	Component 4	Component 5	Component 6	Component 7	Component 8	Component 9	Component 10	Component 11	Total
Ahmed et al [11]	Yes	Yes	Yes	Yes	Yes	Yes	No	Yes	No	Yes	No	High
Alharbi et al [12]	Yes	Yes	Yes	Yes	Yes	Yes	No	Yes	No	No	No	Moderate
Almatrafi et al [13]	Yes	Yes	Yes	Yes	Yes	Yes	No	Yes	No	No	No	Moderate
Atabi et al [14]	No	Yes	Yes	Yes	Yes	Yes	No	No	No	No	No	Moderate
Damiao et al [15]	Yes	Yes	No	No	Yes	Yes	No	No	No	No	No	Moderate
Fakkar et al [16]	Yes	Yes	No	No	Yes	Yes	No	No	No	No	No	Moderate
Gundu et al [17]	Yes	Yes	Yes	No	Yes	Yes	No	No	No	No	No	Moderate
Iftikhar et al [18]	Yes	Yes	Yes	Yes	Yes	Yes	No	No	No	No	No	Moderate
Kim et al [19]	Yes	Yes	Yes	No	Yes	Yes	No	Yes	No	No	No	Moderate
Ko et al [20]	Yes	Yes	Yes	Yes	Yes	Yes	No	Yes	No	Yes	No	High
Liu et al [21]	Yes	Yes	Yes	No	Yes	Yes	No	No	No	No	No	Moderate
Martin et al [22]	Yes	Yes	Yes	No	Yes	Yes	No	Yes	No	No	No	Moderate
Sato et al [23]	Yes	Yes	No	Yes	Yes	Yes	No	No	No	No	No	Moderate
Shahwan et al [24]	Yes	Yes	Yes	No	Yes	Yes	No	Yes	No	No	No	Moderate
Singh et al [25]	Yes	Yes	Yes	No	Yes	Yes	No	Yes	No	No	No	Moderate
Wai et al [26]	Yes	Yes	Yes	No	Yes	Yes	No	Yes	No	No	Yes	Moderate
Yakubu et al [27]	Yes	Yes	No	No	Yes	Yes	No	No	No	No	Yes	Moderate

Effect of Metformin on Deficiency of Vitamin B12 in Patients With T2DM

**Figure 2:**
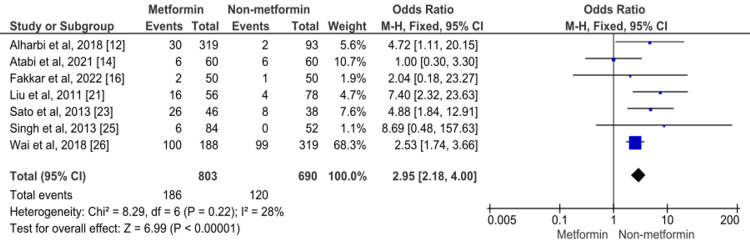
Forest plot showing the association of metformin with vitamin B12 deficiency Sources: References [12,14,16,21,23,25-26]

Overall seven studies assessed the impact of metformin deficiency of vitamin B12 in patients with T2DM. The pooled rate of deficiency of vitamin B12 in patients receiving metformin (23.16%) was significantly higher compared to patients who were not on metformin (17.4%) (OR: 2.95, 95% CI: 2.18-4.00, p-value: 0.001) as shown in Figure 2. No significant heterogeneity was found among the study results (I-square: 28%, p-value: 0.22).

Risk Factors Associated With Deficiency of Vitamin B12 in Patients With T2DM and Receiving Metformin

Table 3 shows the number of studies that assessed each factor associated with deficiency of vitamin B12 in patients with T2DM and receiving metformin along with pooled estimates. For proton pump inhibitors, papers were harmonized according to the use of proton pump inhibitors versus not. Results showed that no significant association is there between proton pump inhibitors and deficiency of vitamin B12. No significant heterogeneity was reported among the study results (I-square: 0%). Studies did not find any significant association between the duration of diabetes and vitamin B12 deficiency.

**Table 3:** Relationship of independent variables with deficiency of vitamin B12 in patients with T2DM and receiving metformin OR: Odds ratio CI: Confidence interval

Factors	Included Studies	Categories	OR (95% CI)	P-value	I-square
Proton Pump Inhibitors (No vs Yes)	5	No	1		
		Yes	0.94 (0.74-1.20)	0.623	0%
Duration of metformin use	3	< 4 Years	1		
		4-10 Years	2.54 (1.22-8.54)	0.001	81%
		<10 Years	2.32 (1.27-4.22)	0.001	91%
Duration of diabetes	4		0.95 (0.89-1.01)	0.113	0%
Daily dose of metformin	4	<1000 mg	1		
		1000-2000 mg	2.58 (1.73-3.83)	0.001	0%
		>=2000 mg	5.89 (2.63-13.19)	0.001	65%

Studies that assessed the duration of metformin use compared patients who used metformin for <4 years with patients who were taking metformin between 4 and 10 years and more than 10 years. Results showed that the odds of deficiency of vitamin B12 are significantly higher in patients with a duration of metformin use of 4-10 years (OR: 2.54, 95% CI: 1.22-8.54, p-value: 0.001) and more than 10 years (OR: 2.32, 95% CI: 1.27-4.22, p-value: 0.001).

Lastly, patients who were taking a dosage of less than 1000 mg per day are less likely to develop a deficiency of vitamin B12 as compared to individuals taking a daily dose of 1000-2000 mg (OR: 2.58, 95% CI: 1.73-3.83, p-value: 0.001) and individuals taking a daily dose of >2000 mg (OR: 5.89, 95% CI: 2.63-13.19, p-value: 0.001)

Discussion

In the current meta-analysis, we evaluated the effect of metformin on the deficiency of vitamin B12 in patients with T2DM. We also examined four factors associated with a deficiency of vitamin B12 in patients with T2DM and receiving metformin, including the use of proton pump inhibitors, duration of metformin use, duration of diabetes, and a daily dose of metformin.

Diabetes is a chronic condition that can have serious, life-threatening complications. When compared to the long-term implications of inducing vitamin B12 deficiency, metformin, a regularly used first-line therapy strategy, has benefits [3]. However, the prevalence of the deficiency of vitamin B12 associated with the usage of metformin in T2DM is affected by certain factors. The current meta-analysis found that a deficiency of vitamin B12 is more common in patients receiving metformin compared to their counterparts. Because metformin delays the absorption of glucose, it has an impact on bacterial overgrowth and small bowel motility [28]. According to the review conducted by Liu et al., metformin reduced vitamin B12 levels in patients with diabetes [29]. The review also found that gastrointestinal adverse events were the frequently observed adverse events in patients with metformin. The reduction in B12 absorption caused by metformin may be the result of digestive alterations that cause the binding of the B12-intrinsic factor (IF) complex [30].

The clinical importance of biochemical change in the concentration of serum vitamin B12 remains controversial. A Greek cohort of 600 diabetes patients revealed a link between vitamin B12-dependent megaloblastic anemia and metformin treatment [31]. Metformin may potentially hasten cognitive decline and the development of diabetic peripheral neuropathy in a way that is dependent on vitamin B12 [5]. Thus, it was implied that deficiency in vitamin B12 concentration should not be overlooked.

In the current meta-analysis, the association between the occurrence of vitamin B12 deficiency and the use of metformin showed a clear dose-dependence relationship, and an association related to the duration of metformin use was found. In the current meta-analysis, we found that doses higher than 1000 mg have demonstrated an independent association with the deficiency of vitamin B12. In a case-control study, one of the important risk factors for the deficiency of vitamin B12 was the use of a high metformin dose [31]. In some studies, the duration of metformin use has also been shown as a risk factor for deficiency of vitamin B12 [31].

Different countries around the world have unique nutrition supplements and eating habits and the prevalence of deficiency of vitamin B12 varies with population and the B12 cut-off utilized. The Institute of Medicine (IOM) recommended that the dose of vitamin B12 may not be adequate for people taking metformin, according to a national survey from the United States that found routine vitamin B12 supplementation may not be able to reverse the biochemical B12 reduction in diabetic patients taking metformin [32]. However, certain studies have found that treatment with vitamin B12 at a higher dose could reverse the deficiency of vitamin B12 [33-34]. Moreover, it is important to check the serum vitamin B12 level of patients with T2DM and receive a greater dose of metformin even if patients do not show any symptoms of vitamin B12 deficiency to prevent diabetic neuropathy and other complications.

The current meta-analysis has certain limitations. First, the examining methods of vitamin B12 concentration were diverse in the included studies. Second, in certain excluded studies, the change in serum vitamin B12 concentration was assessed instead of the deficiency of vitamin B12. Even though factors significantly associated with the deficiency of vitamin B12 are not modifiable, these results can be informative in developing multifactorial, targeted interventions to provide the support and education needed to prevent a deficiency of vitamin B12 in patients with T2DM and receiving metformin.

## Conclusions

In conclusion, our meta-analysis found that the prevalence of vitamin B12 deficiency is greater in patients receiving metformin compared to patients who did not receive metformin. Moreover, factors associated with a deficiency of vitamin B12 in patients with T2DM and receiving metformin included a daily dose of metformin and years of metformin use. Given the importance of vitamin B12 in nutrition, metformin-induced B12 decrease may be harmful to patients with T2DM. Supplemental vitamin B12 may be advantageous for those on metformin. Secondly, it is important to test vitamin B12 levels in patients receiving metformin even if patients are asymptomatic in order to identify deficiency earlier and prevent complications.
